# Zinc Source and Concentration Altered Physiological Responses of Beef Heifers during a Combined Viral-Bacterial Respiratory Challenge

**DOI:** 10.3390/ani11030646

**Published:** 2021-03-01

**Authors:** Paul Rand Broadway, Jeffery Carroll, Nicole Burdick Sanchez, Alyssa Word, Shelby Roberts, Emily Kaufman, John Richeson, Mike Brown, Ken Ridenour

**Affiliations:** 1 USDA-ARS Livestock Issues Research Unit, Lubbock, TX 79403, USA; jeff.carroll@usda.gov (J.C.); nicole.sanchez@usda.gov (N.B.S.); 2 Department of Animal and Food Sciences, Texas Tech University, Lubbock, TX 79409, USA; alyssa.word@cactusfeeders.com; 3 Department of Agricultural Sciences, West Texas A&M University, Canyon, TX 79016, USA; sroberts@alltech.com (S.R.); emily.louann.kaufman@gmail.com (E.K.); jricheson@wtamu.edu (J.R.); 4 Global Animal Products, Inc., Amarillo, TX 79118, USA; msbrown9090@gmail.com (M.B.); kridenour@globalanimalproducts.com (K.R.)

**Keywords:** health, respiratory disease, zinc

## Abstract

**Simple Summary:**

Bovine respiratory disease is one of the greatest health challenges cattle producers face and is most commonly treated with antibiotics. With the current push to reduce the use of antibiotics in livestock production, producers are looking at non-pharmaceutical alternatives such as nutritional supplements. This study aimed to determine if different forms of zinc supplementation could reduce some of the negative health effects associated with bovine respiratory disease. Overall, cattle supplemented with ZinMet (zinc methionine/organic zinc) responded better during the disease as evidenced by blood parameters, decreased lesion severity, and decreased fever. Conversely, cattle fed a large dose of zinc sulfate (inorganic zinc) displayed a higher fever and blood parameters that indicated a greater sickness response. Findings from this study suggest that the type and amount of zinc fed to cattle may influence their response to bovine respiratory disease.

**Abstract:**

To determine the effects of zinc supplementation on the immune response to a combined viral-bacterial respiratory disease challenge, thirty-two beef heifers (255 ± 15 kg) were subjected to a 30-d period of Zn depletion, then randomly assigned to one of three treatment diets fed for 30 d before the challenge: (1) supplementation with 100 mg of Zn from Zn sulfate/kg of DM (Zn100), (2) supplementation with 200 mg of Zn from Zn sulfate/kg of DM (Zn200), and (3) supplementation with 80 mg of Zn/kg of DM from zinc methionine and 20 mg of Zn from Zn sulfate/kg of DM (ZinMet). After the 30-d supplementation period, all heifers were fitted with indwelling vaginal temperature (VT) devices and intra-nasally challenged with 1 × 10^8^ PFU bovine herpesvirus-1 on d -3, and then allowed to rest in outdoor pens for 3 d. On d 0, each heifer was challenged intra-tracheally with an average dose of 2.38 × 10^7^ CFU *Mannheimia haemolytica* (MH), fitted with an indwelling jugular catheter, and then moved into individual stalls in an environmentally-controlled enclosed barn. Whole blood samples were collected at 1-h (serum) and 2-h (complete blood counts) intervals from 0 to 8 h, and at 12, 24, 36, 48, 60, 72, 168, and 360 h relative to MH challenge. Data were analyzed using the MIXED procedure of SAS specific for repeated measures with fixed effects of treatment, time, and their interaction. There was a treatment effect (*p* < 0.01) for VT such that Zn200 heifers had greater VT than Zn100 and ZinMet heifers. There was a trend (*p* = 0.10) for a serum cortisol treatment effect with Zn100 heifers having greater cortisol than ZinMet heifers. Total leukocytes and lymphocytes were greater (*p* ≤ 0.01) in Zn100 heifers than Zn200 and ZinMet heifers, whereas monocytes were less (*p* = 0.05) in ZinMet heifers than Zn100 and Zn200 heifers. Concentrations of IL-6 were greater (*p* = 0.02) in ZinMet heifers than Zn100 and Zn200 heifers. Concentrations of IFN-γ were greater in Zn200 heifers than ZinMet heifers at 0 h, and Zn100 heifers from 0 to 12 h post-MH challenge (treatment x time *p* = 0.02). Serum haptoglobin was not affected by treatment or treatment x time (*p* ≥ 0.36) but increased over time (*p* < 0.01) in all groups. There was a trend (*p* = 0.11) for ZinMet heifers to have less severe nasal lesion scores than Zn100 heifers. The observed differential physiological responses in this study indicate that zinc source and concentration may alter the response to a bovine respiratory challenge in heifers.

## 1. Introduction

Bovine respiratory disease (BRD) is the leading cause of morbidity and mortality in United States feedlots [1], and supplementation of zinc has been identified as a possible means of stimulating immune function when an outbreak of BRD is possible [2]. Whereas performance and carcass results of feeding zinc in excess of nutrient requirements in beef cattle finishing diets have been variable [3,4], zinc has long been associated with immunity in vitro in murine and human models [5]. Specifically, zinc has been reported to affect neutrophil [6], monocyte [7], and T lymphocyte [8] function as well as increase the response of certain pro-inflammatory cytokines [9]. Zinc source, specifically inorganic or organic zinc, may affect the bioavailability and metabolism of zinc post-absorption [10]. Inorganic zinc supplements are typically a zinc product coupled with an inorganic salt, and organic zinc is bound to an organic substance. Chelated forms of zinc, specifically the methionine form, are more bioavailable than the sulfate form [11], and zinc methionine has been reported to enhance the recovery rate of cattle to a viral challenge compared to zinc oxide [12].

Current beef National Research Council recommendations for zinc concentration are 30 mg/kg of DM; however, the most recent feedlot nutritionist survey reported that nutritionists were formulating between 34 and 130 mg/kg DM of zinc with a mean of 87.3 mg/kg [13], whereas the EU limits zinc supplementation to 120 mg/kg. Additionally, Galyean et al. [14] reported decreased morbidity in receiving calves supplemented with 70 mg/kg zinc sulfate or zinc methionine compared to 35 mg/kg, suggesting an immunological advantage of zinc. The objective of the current study was therefore to determine the effects of zinc source and concentration as similarly fed to feedlot cattle in the USA on the physiological and immunological responses of beef heifers to a combined viral-bacterial immunological challenge. The working hypothesis of the research project was that organic zinc (ZinMet) may mitigate some of the negative effects associated with BRD in comparison to inorganic zinc.

## 2. Materials and Methods

### 2.1. Animal Care

All experimental procedures were in compliance with the Guide for the Care and Use of Agricultural Animals in Research and Teaching and approved by the Institutional Animal Care and Use Committee of the Livestock Issues Research Unit (IACUC #1601F).

### 2.2. Experimental Design

Initially, procured heifers (*n* = 125; body weight (BW) 272 ± 3.4 kg) of similar breed type and weight were transported to a preconditioning lot for the acclimation phase. Heifers were processed in accordance with the prescribed procedures of the feedlot, which included a 5-way modified live vaccination for respiratory disease without the inclusion of a bacterin for *M. haemolytica* (MH). Heifers (*n* = 79) that were not treated for illness or lameness were then transported to Amarillo, Texas, and placed on pasture for ~30 days with no access to supplemental zinc. Following the grazing period, heifers were weighed and allocated to 1 of 3 treatments corresponding to a colored ear tag: 1) pelleted starter ration with inorganic Zn incorporated at 100 mg Zn/kg of DM from zinc sulfate (control; Zn100); 2) pelleted starter rations with inorganic Zn incorporated at 200 mg Zn/kg of DM from zinc sulfate (Zn200); and 3) pelleted starter ration with a chelated zinc methionine incorporated at 80 mg Zn/kg of DM from ZinMet (ZinMet^®^ Global Animal Products, Inc., Amarillo, TX, USA) and 20 mg Zn/kg of DM from zinc sulfate (ZinMet). The Zn100 treatment was selected to serve as the positive control as this concentration of zinc supplementation is similar to that which is fed by producers in the U.S. Cattle placed in each of these treatments were balanced for uniformity of body weight, phenotype similarity, and temperament. Cattle with previous treatment history or those displaying clinical signs of illness were excluded from the study at this point. Cattle were weighed again 16 d after treatment application, and a blood sample was obtained via jugular venipuncture. Serum was isolated and sent to the Texas Veterinary Medical Diagnostics Laboratory in Amarillo, TX, for quantification of titers specific for Bovine Herpes Virus-1 (BHV-1). Based on these results and the aforementioned selection protocols, heifers (*n* = 32 total; 11 Zn200, 11 ZinMet, and 10 Zn100) were selected for balance of criteria and transported to the Livestock Issues Research Unit’s Bovine Immunology Research and Development complex in New Deal, TX (~204 km) following 30 d of supplementation.

### 2.3. Challenge Phase

Upon arrival, heifers were allowed to rest overnight with access to fresh water and their respective treatment diets for the entire challenge phase. On d −3, heifers were weighed, fitted with a vaginal temperature (VT) recording device [15], and challenged intra-nasally with approximately 1.0 × 10^8^ plaque-forming units of BHV-1. The virus was reconstituted with sterile saline, and then one syringe of culture containing 1 mL of reconstituted virus was administered per nostril in each heifer using a nasal atomization device. Whole blood and serum were collected via jugular venipuncture to analyze hematology along with other variables. A nasal swab was also collected to evaluate basal BHV-1 shedding. Vaginal temperature devices were programmed to record the temperature every 5 min for the duration of the study. Heifers were monitored for signs of illness following virus administration. On the morning of d 0, heifers were fitted with indwelling jugular catheters, and a nasal swab, whole blood sample, and serum sample were collected. Nasal lesion scores were assigned by evaluation of the coverage of lesions present on the visible naris (1 = 0–25%, 2 = 26–50%, 3 = 51–75%, and 4 = 76–100% lesions present on the visible naris). Following placement of the jugular catheters, heifers were inoculated intra-tracheally with a 100 mL solution of MH at 2.38 × 10^7^ CFU in log phase growth diluted with tryptic soy broth media. The entire process took 10–15 min per animal; therefore, 4 batches of MH were used (8 head/batch) to ensure the maintenance of a fresh culture and leukotoxin concentration. Heifers were placed in 4 time blocks representative of the timing of MH administration and were placed in individual steel bleeding stalls (length 2.28 m, width 0.76m, height 1.67), allowing for normal postural movement and maintenance behaviors in a single climate-controlled building with individual access to water and their respective treatment diets using dividers to prevent cross-contamination. Post-MH inoculation, serum was collected every h for 8 h, every 12 h to 72 h, then on d 7 and d 15, and whole blood was collected every other h for 8 h following MH and at every 12 h to 72 h, then on d 7. Isolated serum was used to quantify concentrations of BHV-1 titers, cortisol, haptoglobin, TNF-α, IL-6, IFN-γ, glucose, and non-esterified fatty acids (NEFAs). Three days post-MH administration, jugular catheters and VT recording devices were removed, and heifers were treated with florfenicol according to label directions and returned to outside holding pens. After sample collection on d 7, heifers were returned to Amarillo, TX, and remained on their respective treatment diets until blood and nasal swabs were collected on d 15.

### 2.4. Laboratory Analyses

Whole blood samples were analyzed for hematology using an IDEXX Procyte Dx Hematology Analyzer (IDEXX Labs, Westbrook, ME, USA) with bovine-specific algorithms. All serum analyses were performed in duplicate. Cortisol was analyzed by a commercially available ELISA kit (Arbor Assays, Ann Arbor, MI, USA). Glucose concentrations were determined by a modification of the enzymatic Autokit Glucose Assay (Wako Diagnostics, Richmond, VA) to fit a 96-well format. Working solution (300 µL) was added to 2 µL of serum or prepared standards in a 96-well plate. Plates were incubated at 37 °C for 5 min, and absorption was measured at 505 nm. Glucose concentrations were determined by comparisons to a standard curve of known glucose concentrations. Similar to glucose, NEFA concentrations were determined by a modification of the enzymatic HR series NEFA-HR assay (Wako Diagnostics, Richmond, VA) to fit a 96-well plate format. Pro-inflammatory cytokines (TNF-α, IL-6, and IFN-γ) were measured in duplicate by a custom bovine multiplex sandwich-based chemiluminescense ELISA kit (Searchlight-Aushon BioSystems, Inc., Billerica, MA, USA).

### 2.5. Statistical Analysis

Data were analyzed using the Proc MIXED procedure of SAS (v.9.4, SAS Institute, Cary, NC, USA) with the fixed effects of treatment, time, and their interactions specific for repeated measures. Vaginal temperature data were summarized into 1-h intervals prior to analysis. Nasal lesion scores and titer data were log transformed to achieve normality prior to statistical analysis. Differences in Least Squares (LS) means were separated at α = 0.05 using the Tukey option.

## 3. Results and Discussion

**Figure 1:**
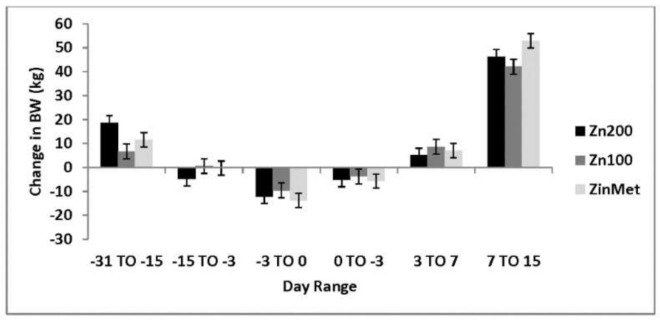
Effect of zinc concentration and source on the change in body weight (BW) of heifers prior to and during a combined viral (d -3)/ bacterial (d 0; MH) respiratory challenge following 30 d supplementation. Zn100 = 100 mg Zn/kg of DM from zinc sulfate, Zn200 = 200 mg/kg of DM from zinc sulfate, ZinMet = 80 mg/kg of Zn/kg of DM from zinc methionine and 20 mg of Zn/kg of DM from zinc sulfate. Treatment: *p* = 0.79; Time: *p* < 0.01; Treatment time: *p* = 0.07.

The average body weight (BW) of heifers at study initiation (d -31) was 272 ± 3.4 kg. There was a tendency (*p* = 0.06) for Zn100 heifers to be heavier than the other treatments for the duration of the study. All heifers lost weight during the challenge phase of the study as expected; however, there were no differences in BW loss between treatments (*p* > 0.05). During the recovery phase, ZinMet heifers gained more weight than Zn100 and Zn200 treatments (*p* = 0.01). These data agree with data by [16] which reported faster recovery in BW in feedlot cattle challenged with BHV-1 and fed zinc methionine compared to inorganic zinc. Overall, there was no difference in the change in BW over the entire study period (*p* = 0.79; Figure 1), but ZinMet heifers, on average, gained more weight compared to Zn100 and Zn200 (51.8, 42.03, and 48.08 kg, respectively). The average daily gain followed the same trend as BW with no overall treatment differences (*p* = 0.87).

Titers for BHV-1 were measured on d −3, 0, 3, 7, and 15. Overall, there were no differences in log titer concentrations (*p* = 0.54) across treatments. One heifer from each of the Zn200 and ZinMet treatments and two heifers from the Zn100 treatment possessed quantifiable BHV-1 titers prior to experimental infection. All animals received a vaccination for BHV-1 ~90 d before the challenge, but the majority of the heifers failed to seroconvert. After the experimental viral administration, titers increased to over 1 log after 3 d and were maintained at > 2.0 log for the duration of the study for each animal, indicating the viral challenge was appropriately administrated and effectively eliciting a response. Nasal shedding of BHV-1 was also monitored at d −3, 0, 3, 7, and 15. Some animals were shedding BHV-1 prior to experimental inoculation, possibly due to a storm prior to the challenge that allowed commingling with other cattle. From d 0 to d 7, viral shedding occurred in over 80% of the heifers and was reduced to less than 10% of the heifers by d 15.

**Figure 2:**
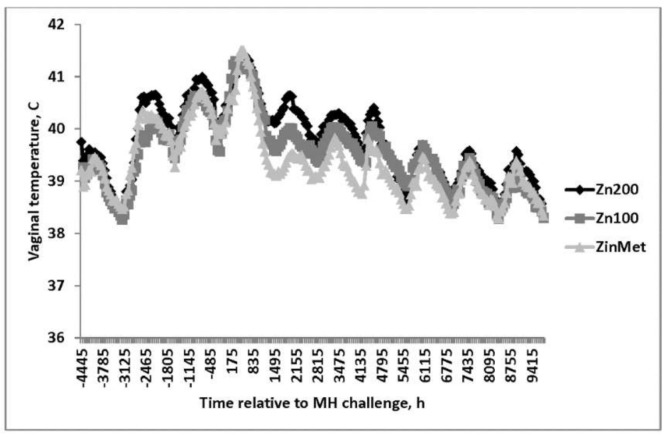
Effect of zinc concentration and source on vaginal temperature of heifers prior to and during a combined viral (d -3)/ bacterial (d 0; MH) respiratory challenge following 30 d supplementation. Zn100 = 100 mg Zn/kg of DM from zinc sulfate, Zn200 = 200 mg/kg of DM from zinc sulfate, ZinMet = 80 mg/kg of Zn/kg of DM from zinc methionine and 20 mg of Zn/kg of DM from zinc sulfate. Treatment: *p* = 0.02; Time: *p* < 0.01; Treatment time: *p* < 0.01; SEM = 0.07.

Vaginal temperature was measured every 5 min throughout the study and was collapsed into 1-h intervals before being analyzed. Overall, Zn200 heifers had the greatest VT following the viral and bacterial challenges (*p* = 0.02; 39.8°, 39.5°, and 39.4° ± 0.07 SEM for Zn200, Zn100, and ZinMet, respectively; Figure 2) in comparison to the other treatments. Temperature spikes were evident following both challenges and remained elevated for ~72 h after the challenge, with peak temperatures greater than 41 °C following the MH challenge. A treatment-by-time interaction (*p* < 0.01) occurred in that after the MH challenge, VT was greater in Zn200 compared to Zn100, and Zn100 had greater temperatures compared to ZinMet. Vaginal temperature in the ZinMet treatment returned to baseline faster than Zn100 and Zn200 heifers. [16] also reported decreased rectal temperature changes in zinc-supplemented feedlot cattle following a viral respiratory challenge.

**Figure 3:**
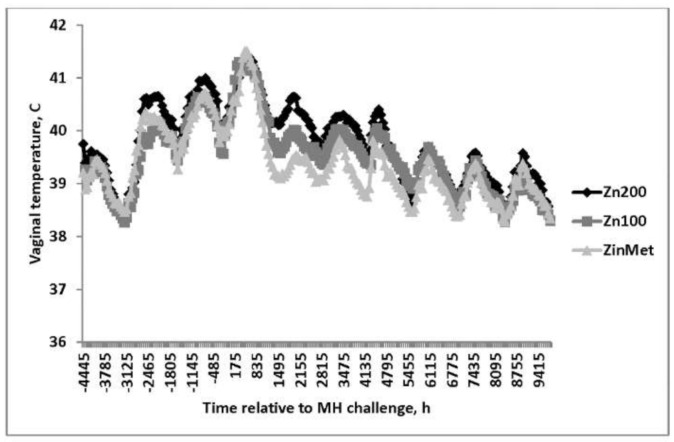
Effect of zinc concentration and source on white blood cells (WBC) of heifers prior to and during a combined viral (d -3)/ bacterial (d 0; MH) respiratory challenge following 30 d supplementation. Zn100 = 100 mg Zn/kg of DM from zinc sulfate, Zn200 = 200 mg/kg of DM from zinc sulfate, ZinMet = 80 mg/kg of Zn/kg of DM from zinc methionine and 20 mg of Zn/kg of DM from zinc sulfate. Treatment: *p* < 0.01; Time: *p* < 0.01; Treatment time: *p* = 0.31.

**Figure 4:**
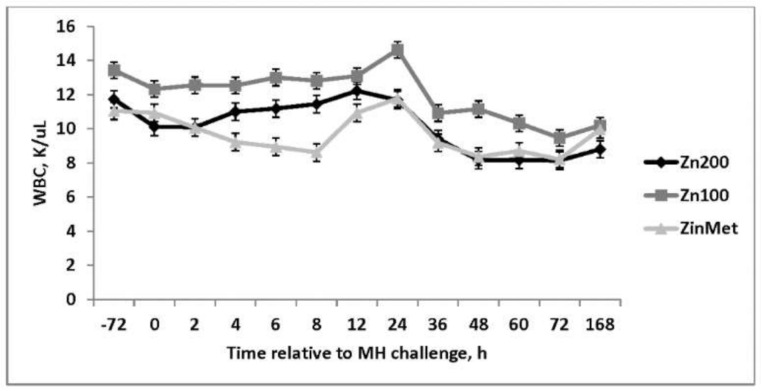
Effect of zinc concentration and source on circulating lymphocyte concentrations of heifers prior to and during a combined viral (d -3)/ bacterial (d 0; MH) respiratory challenge following 30 d supplementation. Zn100 = 100 mg Zn/kg of DM from zinc sulfate, Zn200 = 200 mg/kg of DM from zinc Scheme 80. mg/kg of Zn/kg of DM from zinc methionine and 20 mg of Zn/kg of DM from zinc sulfate. Treatment: *p* < 0.01; Time: *p* < 0.01; Treatment time: *p* = 0.54.

**Figure 5:**
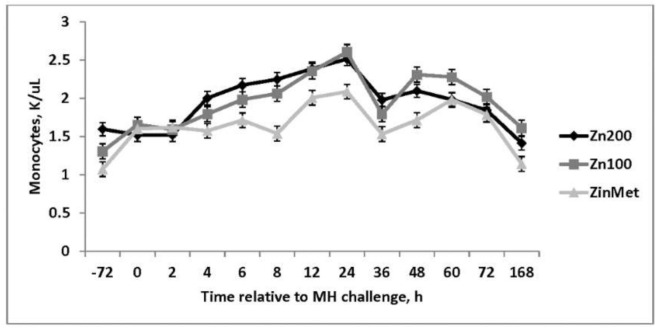
Effect of zinc concentration and source on circulating monocyte concentrations of heifers prior to and during a combined viral (d -3)/ bacterial (d 0; MH) respiratory challenge following 30 d supplementation. Zn100 = 100 mg Zn/kg of DM from zinc sulfate, Zn200 = 200 mg/kg of DM from zinc sulfate, ZinMet = 80 mg/kg of Zn/kg of DM from zinc methionine and 20 mg of Zn/kg of DM from zinc sulfate. Treatment: *p* = 0.05; Time: *p* < 0.01; Treatment time: *p* = 0.22.

There were no treatment differences in overall red blood cells, hemoglobin, neutrophils, or eosinophils (*p* > 0.05). Increased hematocrit was observed in ZinMet heifers compared to Zn100 and Zn200 (*p* < 0.01) throughout the sampling period. Following the viral challenge, there was a decrease in platelets in all treatments from baseline through d 3, and there was a tendency for a treatment-by-time interaction (*p* = 0.07) such that from d −3 to 24 h after the MH challenge, Zn200 > Zn100 > ZinMet but were similar from 48–72 h. Platelets not only contribute to clotting but are also involved with inflammation [17], and the decreased platelets observed in ZinMet heifers may indicate less overall inflammation. An overall treatment effect was observed in white blood cells (*p* < 0.01; Figure 3) where Zn100 heifers were consistently greater than Zn200 and ZinMet heifers through d 7. This same trend was observed in lymphocyte concentrations, likely driving the response observed in overall white blood cells. Lymphocytes were greater throughout the challenge in the Zn100 group (*p* < 0.01; Figure 4). A tendency (*p* = 0.05) for decreased monocytes in ZinMet heifers was observed from d −3 to d 7 (Figure 5) in comparison to Zn100 and Zn200. Therefore, zinc source may have influenced monocyte signal transduction pathways to modulate pathogen sensing receptors. Zinc is known to mitigate infections, both specific and non-specific, by increasing disease resistance. However, supplementation with large amounts of zinc may be detrimental to immune function [5], which was also indicated by greater platelets, and reduced monocytes in Zn200 compared to Zn100 in the current trial.

**Figure 6:**
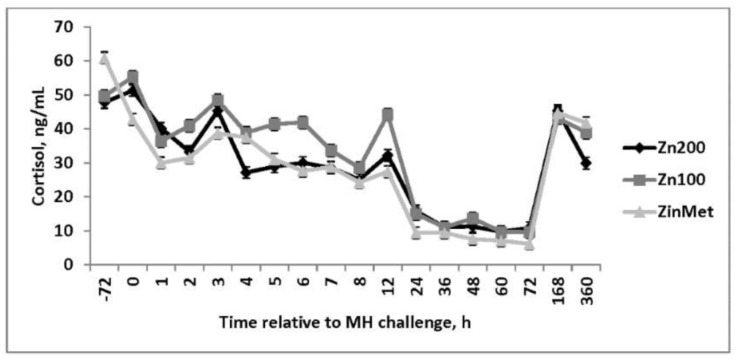
Effect of zinc concentration and source on circulating cortisol concentrations of heifers prior to and during a combined viral (d -3)/ bacterial (d 0; MH) respiratory challenge following 30 d supplementation. Zn100 = 100 mg Zn/kg of DM from zinc sulfate, Zn200 = 200 mg/kg of DM from zinc sulfate, ZinMet = 80 mg/kg of Zn/kg of DM from zinc methionine and 20 mg of Zn/kg of DM from zinc sulfate. Treatment: *p* = 0.10; Time: *p* < 0.01; Treatment time: *p* = 0.36.

**Figure 7:**
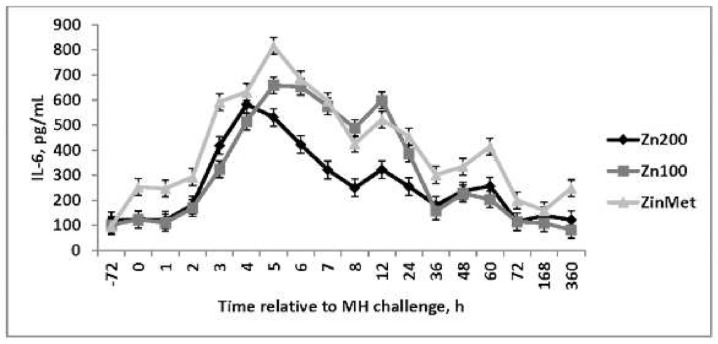
Effect of zinc concentration and source on circulating concentrations of Interleukin-6 (IL-6) of heifers prior to and during a combined viral (d -3)/ bacterial (d 0; MH) respiratory challenge following 30 d supplementation. Zn100 = 100 mg Zn/kg of DM from zinc sulfate, Zn200 = 200 mg/kg of DM from zinc sulfate, ZinMet = 80 mg/kg of Zn/kg of DM from zinc methionine and 20 mg of Zn/kg of DM from zinc sulfate. Treatment: *p* = 0.02; Time: *p* <0.01; Treatment time: *p* = 0.31.

No differences in cortisol concentrations were observed during the BRD challenge (*p* = 0.10; Figure 6). Cortisol increased immediately following the challenge but returned to baseline within 8 h, suggesting acclimation to the bleeding stalls and human interaction. Elevations in cortisol were only present in serum samples collected via jugular venipuncture. No differences (*p* = 0.90) in circulating TNF-α concentrations were observed, and concentrations were decreased in comparison to other immune challenge models such as lipopolysaccharide (LPS) [18]. However, a treatment effect was observed (*p* = 0.02) in IL-6 concentrations (Figure 7) such that ZinMet heifers had increased IL-6 concentrations compared to Zn200 but were similar to Zn100 from 6 to 24 h after the MH challenge. The cytokine IL-6 is an important mediator of fever and is an important regulator in the acute phase response, and this cytokine concentration pattern may partially explain the observed febrile response [19].

**Figure 8:**
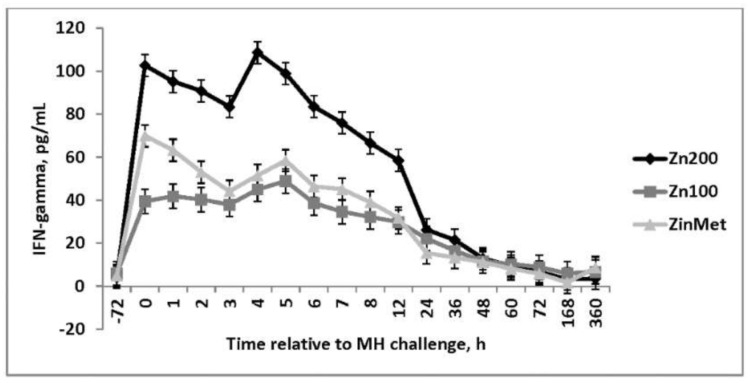
Effect of zinc concentration and source on circulating concentrations of Interfereon gamma (IFNγ) of heifers prior to and during a combined viral (d -3)/ bacterial (d 0; MH) respiratory challenge following 30 d supplementation. Zn100 = 100 mg Zn/kg of DM from zinc sulfate, Zn200 = 200 mg/kg of DM from zinc sulfate, ZinMet = 80 mg/kg of Zn/kg of DM from zinc methionine and 20 mg of Zn/kg of DM from zinc sulfate. Treatment: *p* < 0.01; Time: *p* <0.01; Treatment time: *p* <0.01.

Interferon-γ was elevated in the Zn200 group from 0–12 h following the MH challenge, whereas only minimal differences were observed between the ZinMet and Zn100 treatments (*p* <0.01; Figure 8). This cytokine is critical for innate and adaptive immunity against some viral and some bacterial infections and can directly inhibit viral replication. Interferon-γ induces macrophage activation and may increase MHC I and II expression [20]. Increases in IFN-γ in Zn200 heifers may be the result of increased pathogen load, which complements and further reinforces the hematological parameters measured.

**Figure 9:**
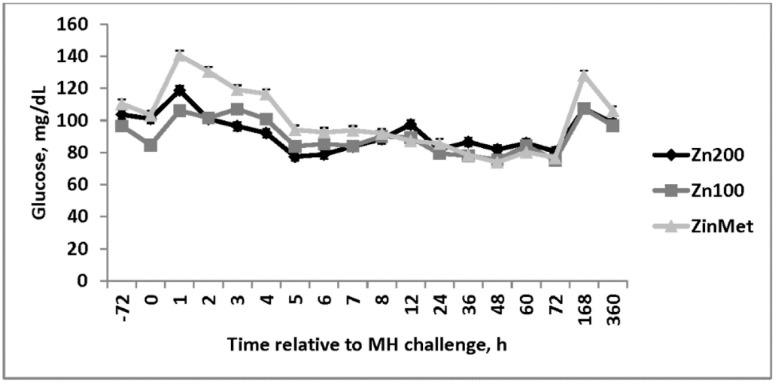
Effect of zinc concentration and source on circulating concentrations of glucose of heifers. bacterial (d 0; MH) respiratory challenge following 30 d supplementation. Zn100 = 100 mg Zn/kg of DM from zinc sulfate, Zn200 = 200 mg/kg of DM from zinc sulfate, ZinMet = 80 mg/kg of Zn/kg of DM from zinc methionine and 20 mg of Zn/kg of DM from zinc sulfate. Treatment: *p* = 0.04; Time: *p* <0.01; Treatment time: *p* = 0.09.

**Figure 10:**
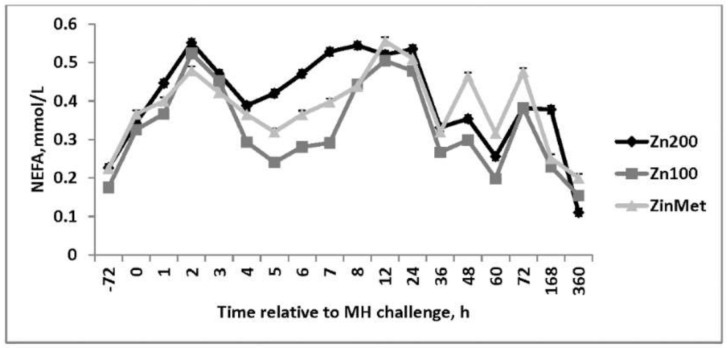
Effect of zinc concentration and source on circulating non-esterified fatty acid (NEFA) concentrations of heifers prior to and during a combined viral (d -3)/ bacterial (d 0; MH) respiratory challenge following 30 d supplementation. Zn100 = 100 mg Zn/kg of DM from zinc sulfate, Zn200 = 200 mg/kg of DM from zinc sulfate, ZinMet = 80 mg/kg of Zn/kg of DM from zinc methionine and 20 mg of Zn/kg of DM from zinc sulfate. Treatment: *p* < 0.01; Time: *p* <0.01; Treatment time: *p* = 0.50.

**Figure 11:**
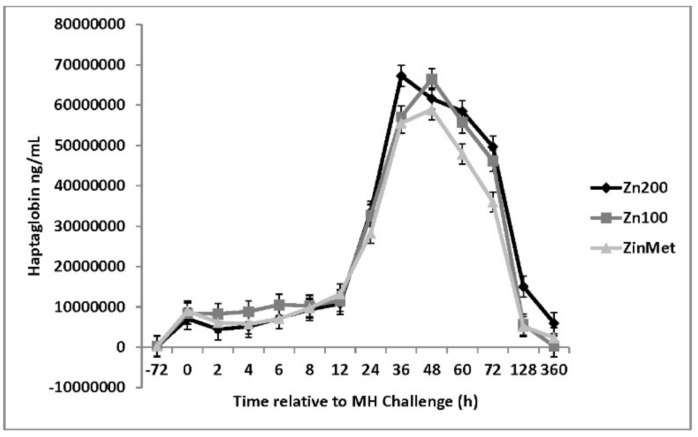
Effect of zinc concentration and source on circulating haptoglobin concentrations of heifers prior to and during a combined viral (d -3)/ bacterial (d 0; MH) respiratory challenge following 30 d supplementation. Zn100 = 100 mg Zn/kg of DM from zinc sulfate, Zn200 = 200 mg/kg of DM from zinc sulfate, ZinMet = 80 mg/kg of Zn/kg of DM from zinc methionine and 20 mg of Zn/kg of DM from zinc sulfate. Treatment: *p* = 0.59; Time: *p* <0.01; Treatment time: *p* = 0.30.

**Figure 12:**
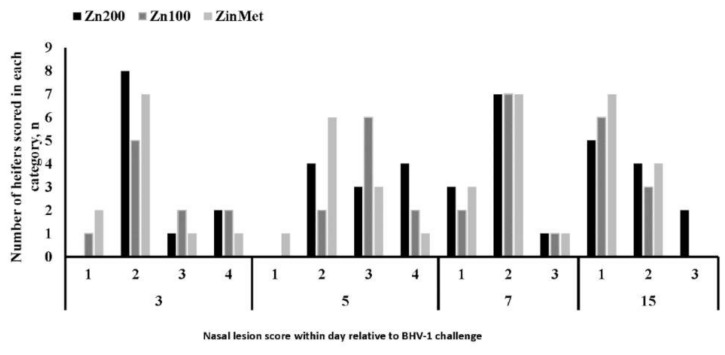
Effect of zinc concentration and source on nasal lesion scores of heifers prior to and during a combined viral (d -3)/ bacterial (d 0) respiratory challenge following 30 d supplementation. Zn100 = 100 mg Zn/kg of DM from zinc sulfate, Zn200 = 200 mg/kg of DM from zinc sulfate, ZinMet = 80 mg/kg of Zn/kg of DM from zinc methionine and 20 mg of Zn/kg of DM from zinc sulfate. Treatment: *p* = 0.11; Time: *p* <0.01; Treatment*time: *p* = 0.11. *p*-values were calculated using log transformed data of nasal lesion severity.

An increase in circulating glucose occurred 1 h post-MH challenge in all treatments. Overall, circulating glucose concentrations were greater in ZinMet heifers compared to Zn100 and Zn200 (*p* = 0.04; Figure 9), suggesting a possible energy sparing effect related to ZinMet. Circulating NEFAs were reduced (*p* < 0.01) in Zn100 compared to other treatments, but there was overall no difference between Zn200 and ZinMet (Figure 10). There was no effect of zinc source on circulating haptoglobin (*p* = 0.59; Figure 11); however, as expected of this acute-phase protein, there was a large increase in haptoglobin occurring 24 h post-MH challenge that lasted until d 7. Nasal lesion scores were measured on d 0, 3, 7, and 15 based on the aforementioned criteria. There was no treatment difference (*p* = 0.11); however, nasal lesions were slightly less severe in the ZinMet heifers (Figure 12), which once again suggested a slight benefit in ZinMet to reduce the severity of illness based on this clinical observation.

## 4. Conclusions

Based upon data from the current study, zinc concentration and source altered some physiological parameters associated with bovine respiratory disease. Overall, ZinMet heifers responded better to the challenge through decreased vaginal temperature, decreased white blood cells, and numerical reduction in the severity of nasal lesions scores. These findings juxtaposed with increased weight gain during recovery suggest the inclusion of chelated zinc may have been the most effective of the treatments tested. Conversely, Zn200 heifers displayed increased vaginal temperature, increased platelets, and greatly increased IFN-γ, suggesting greater disease burden. Therefore, increased supplemental zinc (200 mg/kg of DM) may have been detrimental to the health and well-being of the heifers in comparison to treatments with reduced supplemental zinc inclusion.

## Data Availability

The data presented in this study are available on request from the corresponding author.
